# Synthesis, Structure and VLF Dielectric Permittivity of Hydrophobic, TMOS-Based Silica Aerogels

**DOI:** 10.3390/gels12090796

**Published:** 2026-09-01

**Authors:** Tali Pechersky Savich, Guy Lazovski, Ellen Wachtel, Muriel E. Layani-Tzadka, Adira H. Marcus, Shilat Ashush, Asaf Nissenbaum, Raz Gvishi, Galit Bar, Igor Lubomirsky, David Ehre

**Affiliations:** 1 Department of Molecular Chemistry and Materials Science, Weizmann Institute of Science, Rehovot 761001, Israel; 2 Soreq NRC, Yavne 81800, Israel

**Keywords:** semiconductor devices, parasitic capacitance, hydrophobic silica aerogels, VLF impedance measurements, low dielectric permittivity, interlayer coating

## Abstract

As cutting-edge semiconductor devices become smaller and more densely packed (i.e., ultra large-scale integration, ULSI), there is an increased risk of parasitic capacitance preventing proper device operation. There is consequently growing interest in the development of low-permittivity dielectric materials to serve as intermetal/interlayer coatings that would minimize this effect. Highly porous aerogels exhibit extremely low dielectric permittivity; silica-based aerogel films, in particular, are candidates for this application. The present report focuses on the synthesis, structure and dielectric permittivity of hydrophobic, tetramethyl orthosilicate (TMOS)-based silica aerogels prepared in disc-form. Using our 3D printed polymer sample holder with few metallic components, we are now able to determine the dielectric permittivity of highly porous, hydrophobic silica aerogels in the very low frequency (VLF) range, 10–27 kHz. Commercial systems that operate in the VLF range include circuits for biological signal processing, characterized by low amplitude and frequency, and circuits that operate in marine environments, where the useful frequency range is limited by acoustic signal deterioration. During impedance measurements, aerogel samples were confined in pure, dry oxygen atmosphere. Low mass density (10–250 mg/cm^3^) TMOS-based aerogel discs present structural characteristics and relative dielectric permittivity values in the VLF range that are not readily comparable with analogous data reported to date in the relevant literature.

## 1. Introduction

As cutting-edge semiconductor devices become smaller and more densely packed (i.e., ultra large-scale integration, ULSI), there is greater risk of parasitic capacitance severely hindering proper device operation. Consequently, there is growing interest in low-permittivity dielectric materials as intermetal/interlayer coatings that could minimize this effect. Highly porous aerogels are a two-phase composite of a matrix material (the structural skeleton) and air (or other gas), thereby exhibiting extremely low dielectric permittivity. Aerogel thin films have been widely suggested as suitable candidates for this application [1,2,3,4]. Silica-based aerogel thin films, in particular, have been mentioned in several reports as promising candidates for this application [1,2,3], in part due to the well-established deposition technique using sol–gel technology [5]. Yet, several drawbacks have been noted, including deformation related to shrinkage during drying, non-uniform pore size, and adsorption of moisture, which degrades both the dielectric and mechanical properties [6]. In order to overcome these problems, complex synthetic routes have been proposed, using additives such as elastomers to reduce shrinkage [7], and pre- [8,9] and/or post-drying [10] surface treatments which help to eliminate the effects of moisture, but which, on their own, might deform the aerogel itself. Recent changes in the synthetic protocols are expected to be capable of alleviating most of these drawbacks: (i) by obtaining control over the extent of shrinkage/expansion; and (ii) by modifying the surface functional groups during hot supercritical drying. This is accomplished without the use of additives or the necessity for post-drying procedures. Recent publications in which the broad range of applications for which aerogels may be suited, including electronics, is reviewed, and novel hybrid and/or composite modifications for improving aerogel mechanical properties are described, include [11,12].

Attempts have been made in the relevant literature to systematize the dependence of the experimental dielectric permittivity $\varepsilon_{r}$ of silica-based aerogels on the bulk mass density (ρ) or porosity. However, it is difficult to compare the suitability of models based on data from these two-phase composite systems; sample geometries, synthetic protocols, surface functional groups, fill gas and measurement frequencies vary widely, in addition to the bulk and skeletal mass densities and porosities of the aerogel samples. To cite just a few reports of theoretical or empirically derived models, (i) Fisher et al. [13] described porous aerogel thin films with bulk density ρ > 600 mg/cm^3^,prepared by surfactant templated spin-on deposition (SOD). Measurements combined X-ray reflectometry, spectroscopic ellipsometry and capacitance–voltage at 1 MHz. The films were found to fit the Lorentz–Lorenz model [14] with two adjustable parameters: the relative dielectric permittivity of the skeleton and of the pores. Using an empirical approach to find a correlation between the aerogel relative dielectric permittivity and the bulk mass density ρ, they also found:

(1) $$\varepsilon_{SOD}=(1.02\pm 0.09)+(0.085\pm 0.09)\left[\frac{{cm}^{3}}{g}\right]\rho$$

(ii) Hrubesh et al. [15] conducted measurements of dielectric permittivity at microwave frequencies (2–40 GHz) using a resonance perturbation method with bulk aerogels having pre-baking mass densities ranging from 10 mg/cm^3^ to 500 mg/cm^3^ and following high temperature removal of organic content and water. These data fit the Clausius–Mossotti relation for gases [16]:

(2) $$\varepsilon_{Hrubesh}=1+1.48\left[\frac{{cm}^{3}}{g}\right]\rho$$

(iii) Bruesch et al. [17] conducted measurements using a capacitance bridge at 1 kHz, measuring permittivity for silica aerogels with mass densities ranging from 240 mg/cm^3^ to 830 mg/cm^3^. They found the following correlation:

(3) $$\varepsilon_{Bruesch}=1+1.264\left[\frac{{cm}^{3}}{g}\right]\rho$$

These results suggest that for different synthesis protocols, geometries, porosities and specific frequency ranges measured, a distinct relationship must be established for each aerogel system in order to convincingly model the dielectric properties.

The present report focusses on the synthesis, structure and dielectric permittivity of hydrophobic, tetramethyl orthosilicate (TMOS)-based silica aerogels prepared in the form of small, cylindrically shaped discs rather than thin films. Measurement reproducibility of the aerogel capacitance, obtained via complex impedance measurements, would likely be compromised by surface irregularities, defects, and variation in thickness that are often present in thin films. We have previously described [18] a 3D printed, acrylonitrile butadiene styrene (ABS) polymer fixture, containing a minimum of metallic components, that can be used as a sample holder for impedance measurements on materials with very low dielectric permittivity. (See Supplementary Materials Figure S1) Using this fixture, we are now able to readily determine the relative dielectric permittivity of highly porous, hydrophobic silica aerogels in the very low frequency (VLF) range of 10–25 kHz. Commercial systems that operate in the VLF range include: (i) circuits for biological signal processing, characterized by low amplitude and frequency ≈ few kHz [19,20]; and (ii) circuits that must operate in marine environments, where the useful frequency range is limited by acoustic signal deterioration [21]. During impedance measurements, aerogel samples were held under ambient conditions of temperature and pressure and confined in pure, dry oxygen atmosphere.

## 2. Results and Discussion

### 2.1. Synthesis—Reproducibility and Mass Density Limits

Varying the precursor molar amounts while maintaining the synthesis protocol unchanged throughout the accessible aerogel mass density range is successful in delivering exceptional reproducibility. With the current synthesis recipe, particularly the use of hot SCD in acetonitrile, it was possible to fabricate > 30 batches, (>five aerogels per batch) with almost identical bulk densities and volumetric shrinkage. When the aging time and drying conditions are kept unchanged, aerogel mass density is maintained within a margin of ±2.5 mg/cm^3^, and the variation in volumetric shrinkage is ~±0.6%.

Moreover, the reactivity and solubility of TMOS brought additional benefits to this synthetic approach. As noted above, the concentration of TMOS in the incorporated solution was manipulated by varying the amounts of ethanol and water in each of the original solutions. This enabled the fabrication of aerogels with significantly higher mass densities than previously reported [22], although still limited to ≤250 mg/cm^3^. In order to maintain similar gelation times, the amount of catalyst solution was adjusted to be inverse to the concentration of TMOS. The molar ratio between water and TMOS was kept >4 when possible, to promote three-dimensional cluster formation. [23] The upper limit for this molar ratio was <25. The amount of water was reduced for very low-density aerogels in order to compensate for the increased amount of water that was added as part of the catalyst solution. We note that polymerization under basic conditions can be reversible. [23] As such, at these very low mass densities, even though gelation was observed, it is possible that the gel might dissolve during aging.

### 2.2. Physical Characterization—Bulk Density, Shrinkage Analysis, Optical Transparency, Hydrophobicity

**Figure 1:**
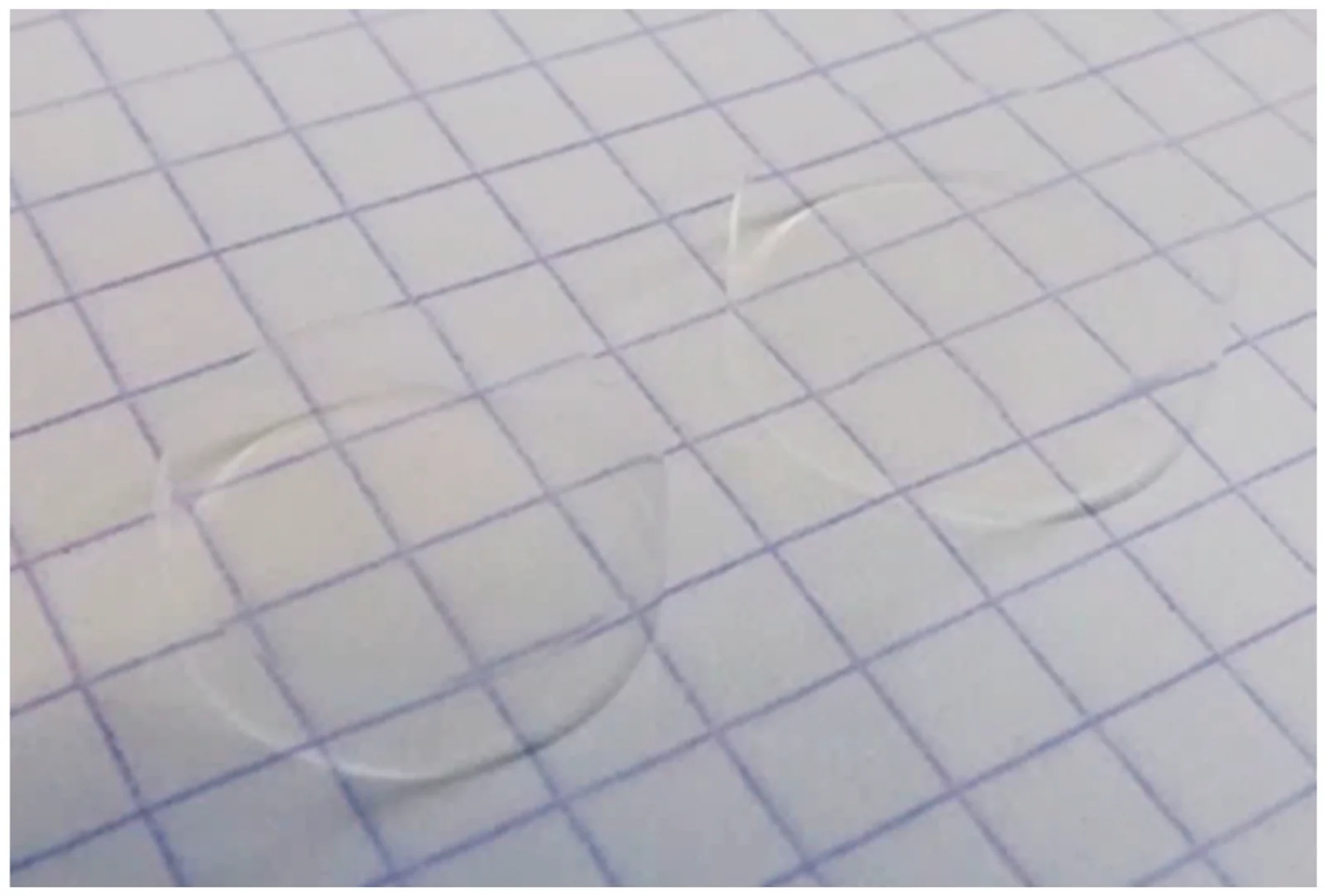
Visual assessment of the optical transparency of two silica aerogel discs prepared in the same batch with TMOS as the precursor. The disc on the left was dried at 45 °C in supercritical CO_2_ with a resulting mass density of 110 mg/cm^3^; the aerogel on the right was subjected to hot supercritical drying with pure acetonitrile as described in Section 4 with a resulting mass density of 80 mg/cm^3^. The right-hand sample is larger in volume due to reduced shrinkage during drying.

Immediately after completion of the SCD process, the samples were removed from their molds, weighed, and dimensions were measured. Using these data, the bulk mass density of each aerogel was calculated, (see Supplementary Materials Table S1). Observed shrinkage may be divided into two regimes. For mass density > 80 mg/cm^3^, the volume shrinkage weakly decreases as the bulk mass density of the sample increases. We attribute this behaviour to the increased strength of the increasingly dense silica skeleton (see also tabulated pycnometry data, Table 1). A similar trend has been reported by Kocon et al. [24]. Below 80 mg/cm^3^, shrinkage decreases sharply as the mass density of the sample decreases. We attribute this effect to the skeletal structure being only partially crosslinked [25]. It appears that the weakened skeleton undergoes expansion opposing the typical shrinkage during drying. This expansion becomes more significant as the target density is further reduced, with 10 mg/cm^3^ aerogel samples observed to almost double in volume during drying. This is due to the abundance of ethanol during gel synthesis and aging, which promotes the formation of ethoxide terminal groups which are less reactive than methoxide or hydroxide termination, and thus hinder the formation of Si-O-Si cross-links. Visual comparison of aerogels synthesized for this work highlights their excellent optical transparency (Figure 1). Although both aerogel discs were prepared in the same batch, different drying protocols—supercritical CO_2_ at 45 °C or hot SCD with pure acetonitrile—influences both mass density and transparency.

**Figure 2:**
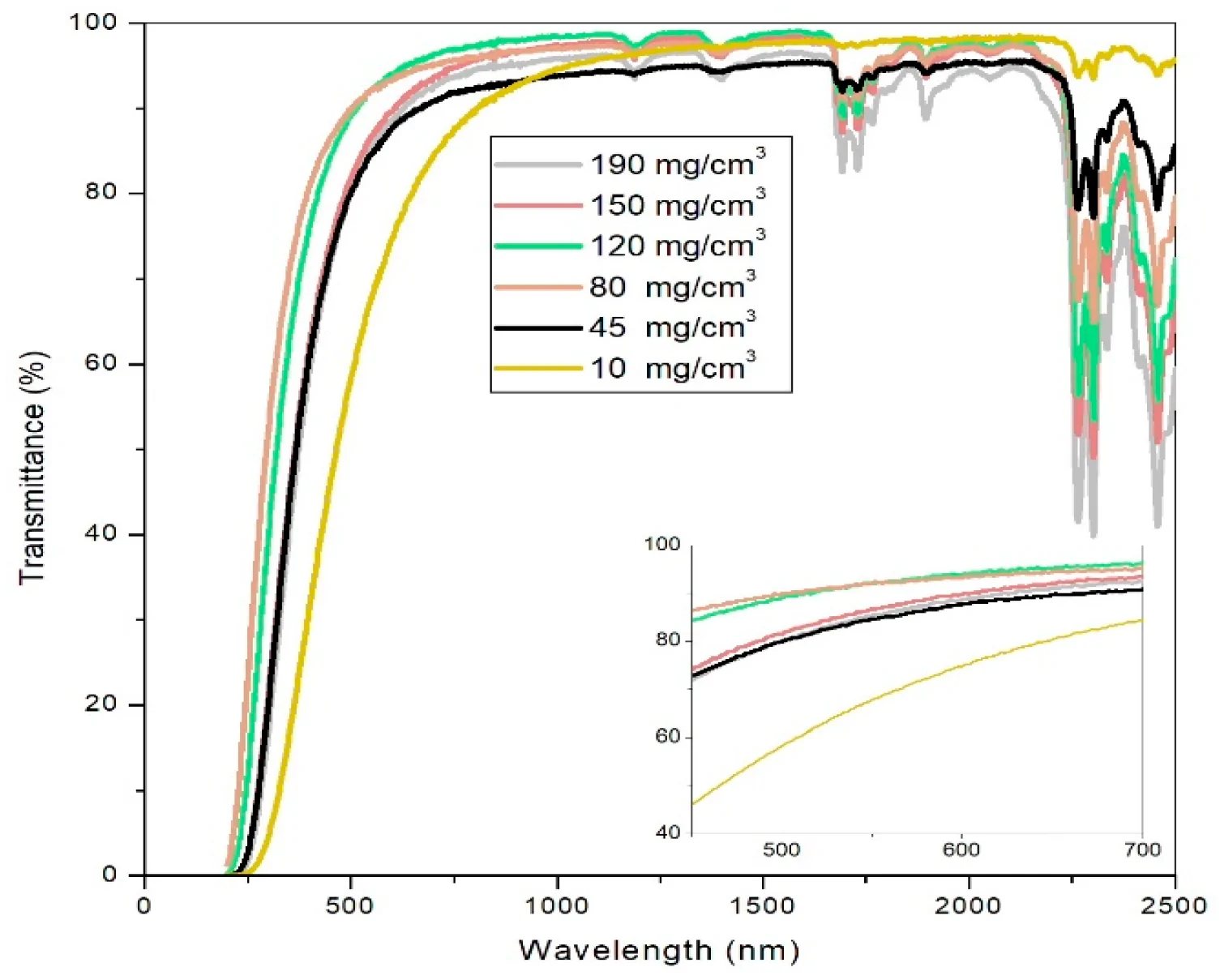
Transmission spectra in the UV–VIS–IR wavelength range measured under ambient conditions for TMOS-based aerogels, prepared as described in Section 4, with mass density between 10 and 190 mg/cm^3^ (see Supplementary Materials Table S4).

Optical transparency was quantified by transmission spectroscopy in the UV–VIS–IR range (Figure 2).

Transmission spectra measured for aerogels of different mass densities demonstrate a broad wavelength range in which sample optical transmittance exceeds 90%. The strong absorption peaks at wavelengths > 1500 nm are due to the ethoxide groups on the silica skeleton which replaced the hydroxide groups during drying. For samples with mass density between 80 and 120 mg/cm^3^, optical transmittance is >90% between 450 and 700 nm (see inset in Figure 2). Very low mass density samples (10–45 mg/cm^3^) display significantly lower optical transparency. Although optical transparency is not currently considered essential for interlayer coatings in ULSI semiconductor devices, the increased use of fiber optics may in the future change that condition.

**Figure 3:**
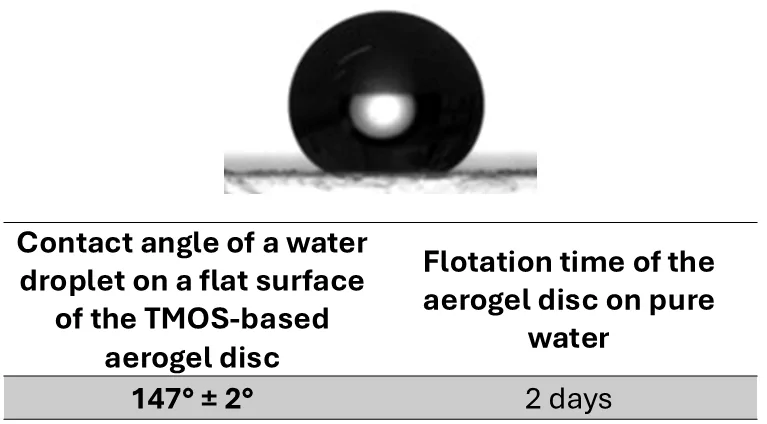
Hydrophobic properties of a TMOS-based aerogel disc, mass density ~90 mg/cm^3^, following hot supercritical drying with pure acetonitrile, are displayed by (1) sessile water drop contact angle measurements, performed as described in Section 4, and (2) flotation time. The water used to form the droplet was pure DDW; the surrounding atmosphere was maintained at STP.

Compared with a number of other silica-based aerogels, for which contact angles below 90° are evidence of a hydrophilic surface, the TMOS-based aerogel disc, prepared as described in Section 4, may certainly be described as being hydrophobic (Figure 3). We note that hydrophobicity is an essential characteristic of interlayers, providing conditions for maintaining surface cleanliness, adhesion, and low surface energy.

**Figure 4:**
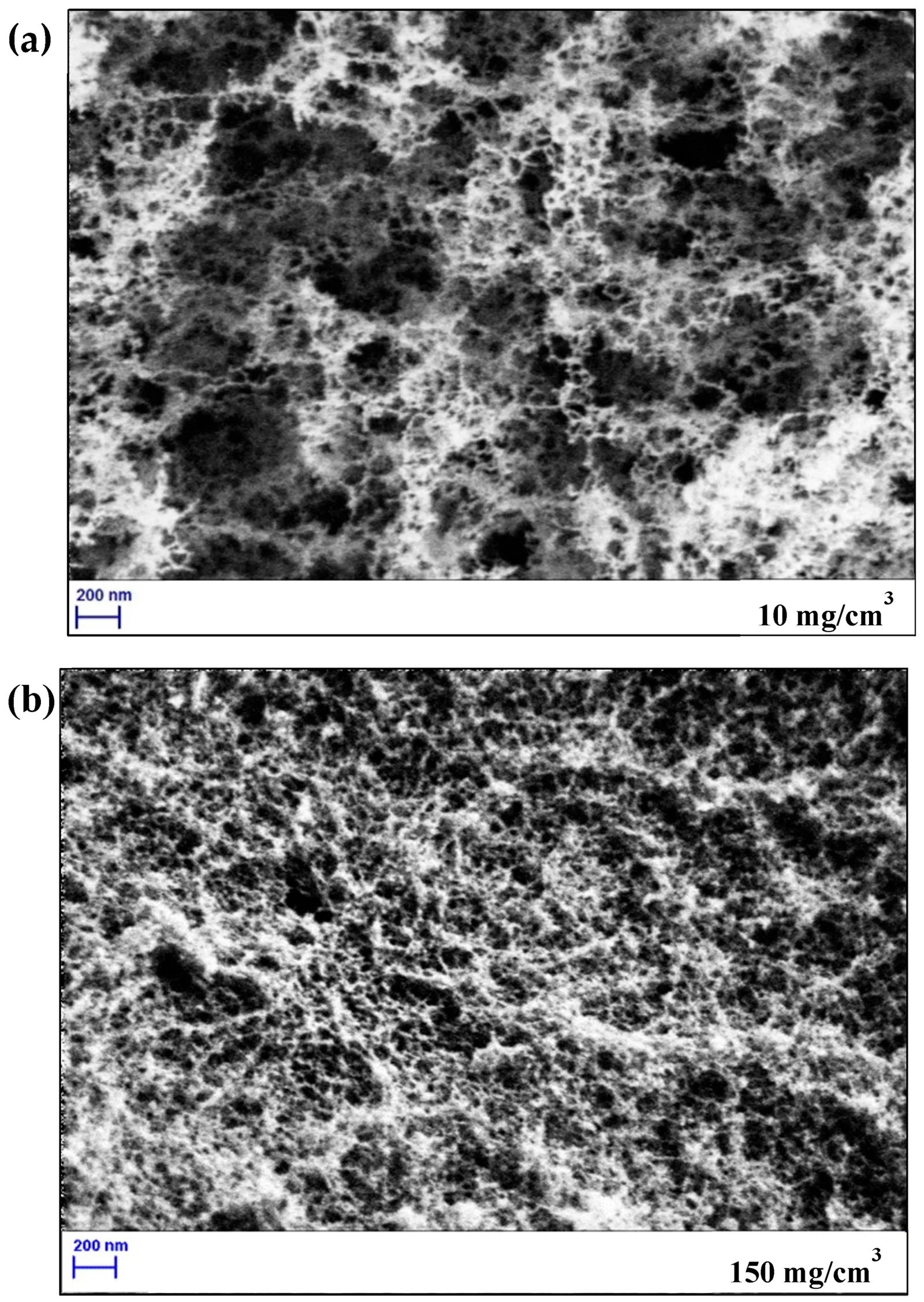
HR-SEM micrographs of (**a**) 10 mg/cm^3^ mass density and (**b**) 150 mg/cm^3^ mass density TMOS-based aerogels prepared as described in the Materials and Methods section. A fracture was scalpel initiated in order to expose the skeletal structure of the bulk material at a surface that had not been affected by the molding protocol, i.e., the surface would not have been interfaced to the Teflon mold nor to the glass cover slide. The scale bar in both panels represents 200 nm. A more complete set of HR-SEM micrographs are available in the Supplementary Materials Figure S2.

We note that an assumed connection between the pore size and the curvature of an adsorbate interface does not apply in the case of a sparse network, such as that of a silica aerogel. It is therefore not correct to analyze nitrogen adsorption isotherms measured for silica aerogels in terms of pore size distributions and/or specific surface area [26]. As shown by HR-SEM imaging (Figure 4), the skeletal structure consists of a network of polymer chains or strings of particles. Adopting the terminology of Ref. [26], the particles are termed “links”, that are joined at junctions, termed “nodes”. We have therefore made pycnometry measurements of the skeleton as a function of mass density of the aerogel discs (Table 1).

The significantly lower mass density of the TMOS skeleton, when compared to that of pure silica, derives from the organic component of the ethoxide terminal groups. On the basis of the pycnometry data, the partial specific volume of pores is estimated to vary from 3.43 cm^3^/g for the most denseaerogel disc to 32.3 cm^3^/g for the least dense.

### 2.3. Complex Impedance Measurements of the Relative Dielectric Permittivity of the TMOS-Based Aerogel Discs

**Figure 5:**
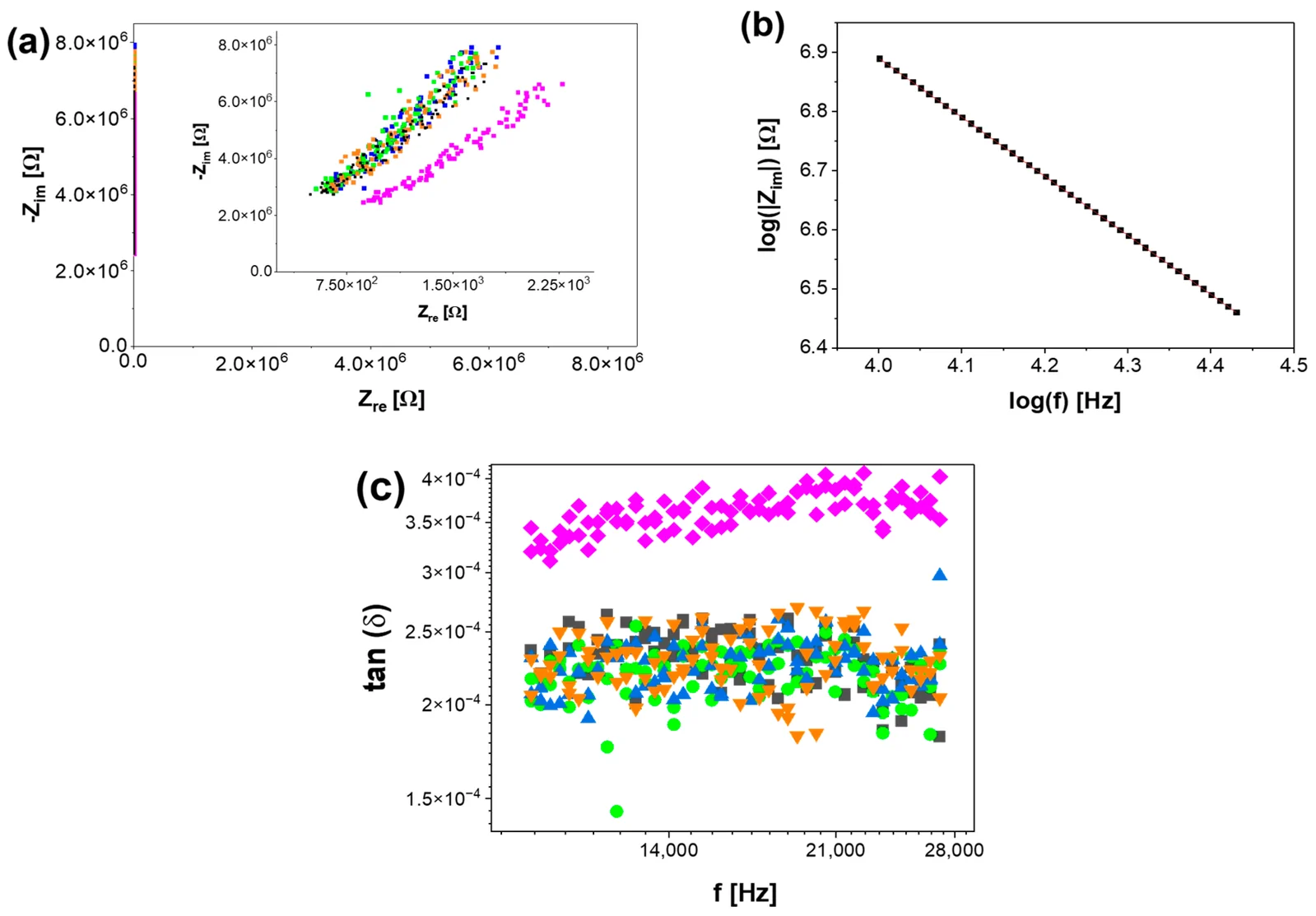
(**a**) Nyquist plots of complex impedance of different mass density aerogel discs, prepared as described in Section 4, measured in dry oxygen atmosphere (99.995% purity), excitation 20 $V_{AC}$, frequency range: 27–10 kHz (duplicate measurements for each sample). Aerogel mass densities: blue—4.92 mg/cm^3^, green—79.88 mg/cm^3^, orange—80.26 mg/cm^3^, black—146.37 mg/cm^3^, pink—15.00 mg/cm^3^. The inset presents an expanded frequency region. (**b**) A representative Bode plot: $log(\left|Z_{im}\right|)$ vs. log(f) for an 80.26 mg/cm^3^ mass density aerogel, measured in oxygen (99.995%) atmosphere. Slope = −0.99929 ± 0.00008, $R^{2}=1$. Bode plots for all aerogels measured are provided in the Supplementary Materials Figure S3. (**c**) Loss tangent (tan *δ*) of the different mass density aerogel discs, prepared as described in Section 4, and measured in dry oxygen atmosphere (99.995% purity), excitation 20 $V_{AC}$, frequency range: 27–10 kHz (duplicate measurements for each sample). Aerogel mass densities: blue—64.92 mg/cm^3^, green—79.88 mg/cm^3^, orange—80.26 mg/cm^3^, black—146.37 mg/cm^3^, pink—215.00 mg/cm^3^.

The complex impedance of TMOS-based hydrophobic aerogels, with mass density ranging from 64.92 mg/cm^3^ to 215.00 mg/cm^3^, was measured. For each sample, Nyquist plots of $-{|Z}_{im}|$ vs. $Z_{re}$ were prepared (Figure 5). The trend observed indicated that lower aerogel mass densities corresponded to higher resistivity. These measurements also confirmed the validity of the assumption that ${|Z}_{im}|$>>$Z_{re}$. As evident from the example in Figure 5b, when plotting $log(|Z_{im}|)$ vs. log(f), the slope is −1, thereby confirming our second assumption that the rate of increase of $\left|Z_{im}\right|$ with frequency *f* is constant ($R^{2}\approx 1$ for all samples). As shown in Figure 5c, the value of the dielectric loss tangent (tanδ) remains below $4.1\times 10^{-4}$ across all synthesized formulations within the 10–27 kHz range. While the aerogel sample with a mass density of 215.00 mg/cm^3^ exhibits a slightly elevated baseline ($\tan\delta \approx 3.5\times 10^{-4}$) due to the presence of more residual polarizable groups, all other samples converge onto a lower loss plateau of $∼2.2\times 10^{-4}$. This behaviour confirms that the baseline dielectric dissipation is consistently suppressed across processing conditions.

For each sample, capacitance was extrapolated using Equation (7), from which we could calculate the dielectric permittivity of each sample (using Equation (9)).

**Figure 6:**
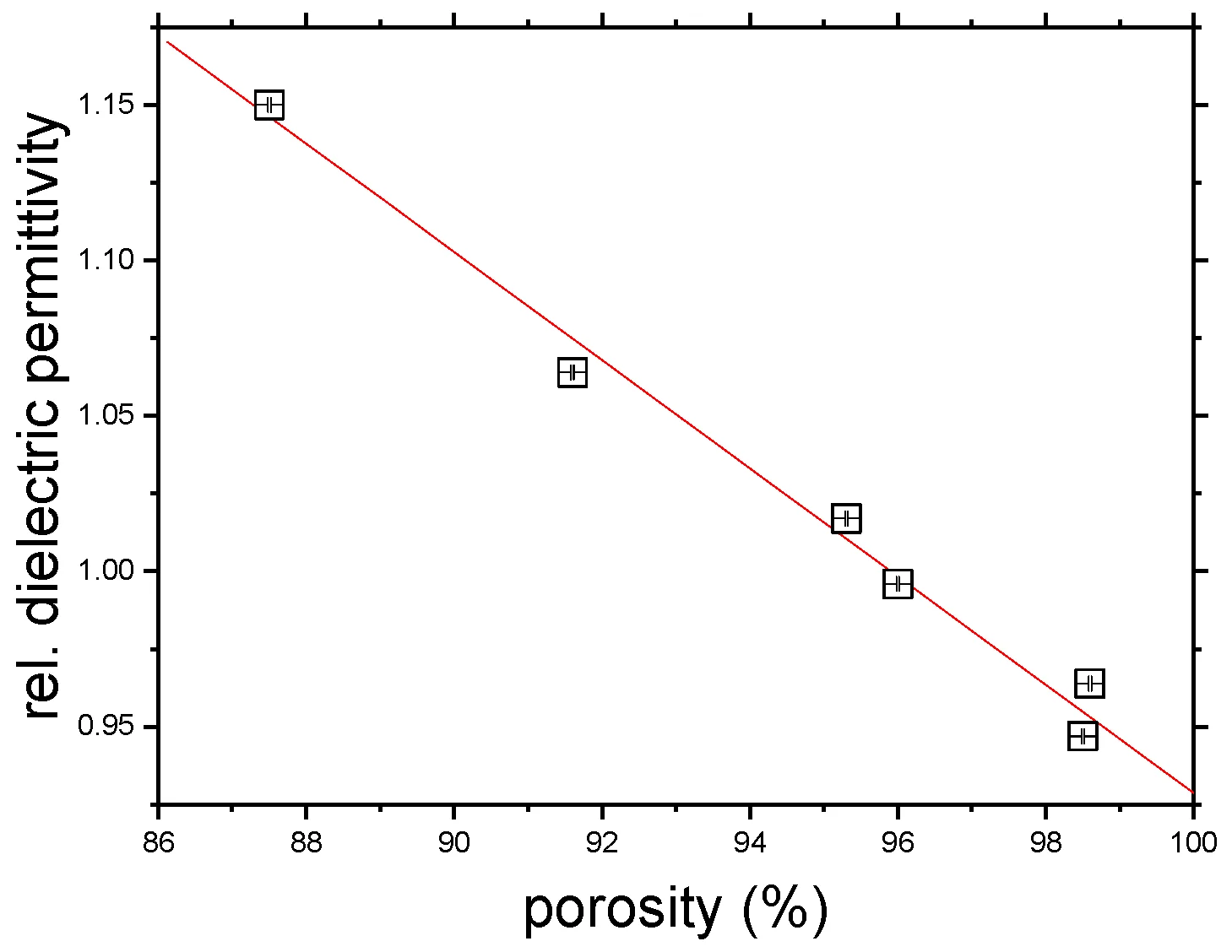
Relative dielectric permittivity in the VLF range, 10 - 27 kHz, calculated from complex impedance measurements in dry oxygen (purity, 99.995%) atmosphere as described, in Section 4 vs. TMOS-based aerogel porosity. ($Porosity=1-\frac{\rho_{A}}{\rho_{S}})$ where $\rho_{A}$ is the aerogel disc mass density and $\rho_{S}$ is the skeletal mass density as determined by pycnometry, tabulated in Table 1.

The calculated relative dielectric permittivity was plotted against the aerogel porosity (Figure 6). Although we measure only a narrow range of aerogel mass densities, approximately linear behaviour is observed for these samples, relating the dielectric permittivity to the porosity.

We also observe in Figure 6 that the calculated relative dielectric permittivity for aerogel discs with porosity > 98% appears to be less than 1, i.e., less than the dielectric permittivity of free space. There are a number of possible explanations for this counterintuitive behaviour. For dielectric capacitors, permittivity is directly proportional to the energy storage capacity of the dielectric. One explanation may be that electrostatic charge is adsorbed on the TMOS-based aerogel surface which is covered with ethoxy functional groups. When comparing surface electrostatic charge (measured as described in Section 4) both before and after insertion into the O_2_ filled zip-lock bag, and before and after complex impedance measurements in O_2_ atmosphere, we observed that the charge on the aerogel samples does in fact change. (See Supplementary Materials Table S5). Aerogel specific surface area is expected to be very large and the static electric susceptibility may indeed result in an effectively lower measured capacitance [27]. An alternative explanation is that the aerogel disc sample diameter is larger than the diameter of the aluminum electrode (see Supplementary Materials Table S3). Examining Equation (8), we find that the calculated relative dielectric permittivity may in fact underestimate the correct value, since not all the fringing field passes through the dielectric; rather, a part, if not the major part, of the fringing field passes through the dry oxygen atmosphere.

## 3. Conclusions

Low mass density (10–250 mg/cm^3^) TMOS-based aerogel discs, synthesized and dried according to the protocols described in Section 4, present structural characteristics and relative dielectric permittivity values in the VLF range (10 - 27 kHz) that are not readily comparable with analogous data reported in the relevant literature. Dielectric permittivity values were calculated from the capacitance of the disc samples as determined by complex impedance measurements performed in dry oxygen atmosphere on a 3D printed fixture as sample holder. Our objective in this report, as well as in the previous description of the polymer fixture [18], has been to simplify to the extent possible preparation protocols, electrical and structural characterization of aerogels that may be effective in minimizing parasitic capacitance in increasingly densely packed electronics. With regard to the particular TMOS-based high porosity samples described here, measurements that remain to be made include dielectric strength, elastic moduli as well as thermal conductivity in order to further determine the suitability of these aerogels for large-scale production and for ULSI interlayer applications.

## 4. Materials and Methods

### 4.1. Materials

Chemicals used in the aerogel synthesis included 99% tetramethyl orthosilicate (TMOS, Sigma-Aldrich, 3 Plaut St. Rehovot 7670603, Israel) as the source of silica, anhydrous ethanol as solvent, and double distilled water for hydrolysis. Ammonium hydroxide solution (28–30 wt%, purity ≥ 99.99%, Sigma-Aldrich) acted as a catalyst upon further dilution with water. Anhydrous acetonitrile (extra dry, purity ≥ 99.5%; Biolabs, Bio-Lab Ltd. P.O.B 34038 Jerusalem 9134001 Israel, was used as the drying solvent. Pure dry oxygen gas was purchased from Maxima Ltd., PO Box 1221310 Haogen St.,Northern Industrial ZoneAshdod 7760049, Israel.

### 4.2. Aerogel Synthesis

Silica aerogels of different mass densities were synthesized via hydrolysis and condensation of TMOS following a procedure similar to the protocol described in [28], using a single step recipe with basic catalysis. A catalyst solution was prepared in advance by mixing 8.20 g ammonium hydroxide solution and 20 mL double distilled water, to be used in all synthetic procedures described below. Prior to each procedure, six cylindrical Teflon molds (3 mm depth and 30 mm diameter; for some samples, 5 mm depth and 18 mm diameter) were placed in a 90 mm diameter glass Petri dish, which would later be used to enclose the filled molds. The aerogel synthesis was carried out as follows. (i) Two solutions were prepared and separately mixed for 5 min in closed, pre-cleaned and dried glass vials. The first solution (A) contained the catalyst, double distilled water and ethanol; the second (B) consisted of TMOS and ethanol. (ii) The solutions were then combined and further mixed for an additional 1.5 min. (iii) The resulting solution was poured into the Teflon molds to the extent that the solution was almost overflowing. The molds were loosely enclosed by covering the large glass Petri dish to minimize evaporation. After approx. 1.5 min, the cover was removed, and each mold was separately covered with a glass slide to obtain a gel with defined cylindrical shape. Gelation took place during a few minutes (see Supplementary Materials Table S1) while the filled molds were maintained under ambient conditions and again enclosed by the large glass Petri dish. The molds were then immersed in an ethanol bath and after approx. 4–5 h, the glass slides were gently removed. The molds were kept immersed in the ethanol bath for at least 12 h following gelation. Sample volume was assumed to be the same as that of the molds as no expansion or shrinkage of the wet gels was observed. The molds were then transferred to the supercritical drying system.

### 4.3. Hot Supercritical Drying (SCD)

Drying of the aerogel discs was performed using a custom-built, “hot” supercritical drying system (Parr Instrument Company, 211 Fifty-Third StreetMoline, IL 61265-1770309-762-7716(800) 872-7720). The drying chamber was partially filled with 180 mL of acetonitrile, and the gels were then transferred from the ethanol bath to the drying chamber. Following leak testing with dry nitrogen gas, the pressure in the drying chamber was brought to 40 bar with added nitrogen, and the drying chamber was heated at 0.5 K·min^−1^ to 295 °C (568 K) with pressure of ≤ 100 bar. After reaching the target temperature, the drying chamber was stabilized for 30 min, followed by pressure release at 0.5 bar·min^−1^, during which time the temperature was kept constant. After reaching 1 bar, the temperature remained unchanged for an additional 4 h while a continuous flow of nitrogen (100 sccm) was used to remove solvent residue from the drying chamber. Finally, the drying chamber was passively cooled to room temperature duringapprox. 10 h. Functional groups remaining on the silica skeleton, when drying at high temperature, are alkoxide groups due to the presence of ethanol within the gel volume. The volume of the dry sample was measured with an Olympus DSX1000 microscope (non-contact, 67-4 Takakura-machi, Hachioji-shi, Tokyo 192-0033 Japan) and with a Fuji digital micrometre (10 μm precision, 520-6 Kamihongo, Matsudo, Chiba, 271-0064 Japan).

### 4.4. Aerogel Structural Characterization

The mass density and volume shrinkage of the aerogel samples following hot SCD were calculated from weight (Sartorius Cubis ultramicro balance MSE2.7S, Otto-Brenner-Straße 20, 37079 Göttingen, Germany) and dimensional measurements as described above. UV–Vis–Near IR transmittance was quantitated using a Jasco V-770 spectrophotometer, 28600 Mary’s Court, Easton, MD 21601, USA. Hydrophobicity was characterized using sessile DDW water droplet contact angle measurements performed using a Ramé-Hart Model 200 Goniometer, 582 W 8360 S, Sandy, UT 84070 USA. Helium pycnometry was performed to determine the mass density of the aerogel skeleton using a Micromeritics AccuPyc 1340 Pycnometer, 4356 Communications DriveNorcross, GA 30093-2901United States. 100–200 purge procedures and 50–100 measurement cycles were performed to ensure measurement reliability. Microstructure was imaged using a high-resolution scanning electron microscope (Zeiss Gemini, Sigma 300 VP HR-SEM, Carl-Zeiss-Promenade 1007745 JenaGermany). SEM measurement conditions: EHT = 1 kV; WD = 2 mm; aperture size = 30 mm; magnification = 70 kX. A fracture in the aerogel disc was initiated with a scalpel in order to expose the skeletal structure at a surface that had not been affected by the molding protocol, i.e., that would not have been interfaced to the Teflon mold nor to the glass cover slide.

### 4.5. Complex Impedance Measurements

The fixture used for measuring complex impedance of the aerogel disc samples was described in detail in Ref. [18] (see also Supplementary Materials Figure S1). Briefly, the fixture was printed using Filamentech™ acrylonitrile butadiene styrene (ABS) polymer filaments on a Flashforge^TM^ Adventurer 3 (wall thickness 0.8 mm, fill density 15%, pattern-hexagons) 3D printer. A disc-shaped aluminum electrode, diameter 25.4 mm, was attached with cyanoacrylate adhesive to a moveable vertical rod, permitting adjustment to the sample thickness. An identical Al disc electrode was glued to the bottom stage. The rod, through which the longest wire runs, was coated with silver paint and grounded to a sub-miniature version A (SMA) connector with a copper wire, effectively enclosing the silver-coated wire in a Faraday cage. The connecting wires for the electrodes were glued with silver epoxy to the aluminum surface and soldered at the opposite end with Sn-Ag-Cu (SAC) alloy to the SMA connectors.

To measure the complex impedance of the aerogel discs, an Alpha Impedance Analyzer (Novocontrol Technologies GmbH & Co) was used, applying 20 $V_{AC}$ excitation voltage within the frequency range 27 kHz to 10 kHz. The electric field was applied perpendicular to the parallel disc electrode configuration, with a sampling interval of 100 data points per frequency decade. Samples were placed between the electrodes, while the top rod was fixed with tape during the measurement to maintain electrical contact between the electrodes and the aerogel. To measure the dielectric permittivity of the aerogels in a dry gas environment, the setup was enclosed in a polyethylene zip-lock bag pre-filled with high-purity, dry oxygen gas (purity ≥ 99.995%). During the impedance measurements, gas temperature was controlled to 23.1–23.7 °C (296.25–296.85 K) and gas pressure was measured to be approx. 0.99 atm. Aerogel samples were measured in duplicate. Electrostatic charging of the aerogel disc samples in oxygen atmosphere was estimated by FMX-003 Electrostatic Fieldmeter (Simco-Ion).

### 4.6. Calculating the Dielectric Permittivity

The relative dielectric permittivity ($\varepsilon_{r}$) of an aerogel disc sample was calculated from impedance measurements as previously reported for fused silica wafers [18]. To summarize the calculation: an equivalent RC circuit was provided for each sample, including a single capacitor and resistor connected either in parallel or in series (similarly to the references [29,30]). For materials with high resistivity and low dielectric permittivity, the imaginary component of the complex impedance ($Z_{im}$) will be much larger than the real component ($Z_{re}$), |$Z_{im}$ | >> $Z_{re}$. Consequently, within a limited frequency range, the capacitance (*C*) for both RC circuit configurations can be estimated as:

(4) $$C\approx \frac{-Z_{im}}{\omega ({Z_{im}}^{2})}=\frac{-1}{\omega {\cdot Z}_{im}}$$

where ɷ=2πf, and *f* is the AC excitation frequency. For a parallel plate disc-shaped capacitor of area *A* and inter-electrode spacing *d*, the ideal geometric capacitance *C_0_* is:

(5) $$C_{0}=\frac{\varepsilon_{0}\cdot A}{d}$$

where $\varepsilon_{0}$ is the dielectric permittivity of free space (8.854 pF/m). The diameter of all aerogel samples was larger than that of the electrode (see Supplementary Materials Table S2) while *A* is set equal to the surface area of the aluminum electrodes in the fixture. When the material between the electrodes is characterized by relative dielectric permittivity $\varepsilon_{r}$ at excitation frequency *f*,

(6) $$\varepsilon_{r}=\frac{C}{C_{0}}\approx \frac{-1}{Z_{im}\cdot \omega \cdot C_{0}}$$

Plotting $log(Z_{im}$) vs. log(f) on a Bode plot, the y-intercept determines the measured capacitance ***C***.

(7) $$\log\left(y_{intercept}\right)=\frac{1}{2\pi \cdot C}\to C=-\frac{1}{2\pi \cdot 10^{\left(y_{intercept}\right)}}$$

Following our previous report [18], we have used a first order approximation, as described in Ref. [31], to correct for the contribution of the fringing field (edge effect) to the sample capacitance:

(8) $$C\approx \varepsilon_{r}C_{o}(1+\frac{d}{\pi R}\ln\left(\frac{2\pi eR}{d}\right))$$

where ***C*** is the measured capacitance; e is Euler’s number; and *R* (=(*A*/*π*)^0.5^) is the radius of the disc electrodes; dividing the measured capacitance by the sum of the ideal capacitance and the capacitance due to the fringing fields we find:

(9) $$\varepsilon_{r}\approx \frac{C}{C_{o}(1+\frac{d}{\pi R}\ln\left(\frac{2\pi eR}{d}\right))}$$

## Figures and Tables

**Table 1:** Mass density of the skeleton as determined by pycnometry of the TMOS-based aerogel discs, prepared as described in Section 4. The mass density of pure silica is approx. 2.2 g/cm^3^.

Aerogel Bulk Density ρ_A_	Skeletal Mass Density ρ_S_	Calculated Porosity 1 − (ρ_A_/ρ_S_)
±5 (mg/cm^3^)	±0.03 (g/cm^3^)	(%)
250	1.76	85.8
175	1.75	90
95	1.73	94.5
75	1.75	95.7
50	1.56	96.7
30	1.48	98.0

## Data Availability

The data supporting the study will be made available from the corresponding author upon reasonable request.

## Supplementary Materials

The following supporting information can be downloaded at: https://www.mdpi.com/article/10.3390/gels12090796/s1, Section S1. 3D printed fixture for impedance measurements; Figure S1. Two views of the 3D printed ABS measurement fixture; Section S2. Protocol for preparing TMOS-based aerogels using hot supercritical drying in pure acetonitrile; Table S1. Gram weight of components and time required for preparation of TMOS-based aerogel discs prior to hot supercritical drying; Table S2. Molar ratios of components input for synthesis of TMOS aerogel discs characterized by volumetric shrinkage following hot supercritical drying and measured mass density; Table S3. Dimensions of aerogel discs following hot supercritical drying; Table S4. Dimensions of (thick) aerogel discs following hot supercritical drying and measured mass density; Section S3. SEM imaging of TMOS-based aerogels; Figure S2. SEM images of TMOS-based aerogels, synthesized as described in the Materials and Methods section of the main text, with mass density ranging from 15 to 150 mg/cm^3^; Section S4. Impedance measurements of TMOS-based aerogels in dry oxygen atmosphere in the VLF range; Figure S3. Bode plots of $Z_{im}$ (complex impedance data measured in duplicate) vs. frequency for TMOS-based aerogels in dry oxygen, mass density 19.76 mg/cm^3^ to 215 mg/cm^3^; Table S5. Electrostatic charging of aerogel disc samples.

## Abbreviations

The following abbreviations are used in this manuscript:

TMOS	Tetramethyl orthosilicate
VLF	Very low frequency
