# Supplementary material for: Synthesis, Structure and VLF Dielectric Permittivity of Hydrophobic, TMOS-Based Silica Aerogels
Source: Gels. 2026 Sep 1;12(9):796. doi: 10.3390/gels12090796 (PMC13606491; doi:10.3390/gels12090796)
Supplement: Supplementary file 1 [file gels-12-00796-s001.zip › gels-4473059-supplementary.pdf]

Supporting Information

for

Synthesis, structure and VLF dielectric permittivity of hydrophobic TMOS-based silica aerogels

Tali Pechersky Savich[1, ‡], Guy Lazovski[2, ‡,*], Ellen Wachtel[1], Muriel E. Layani-Tzadka[2], Adira H. Marcus[2], Shilat Ashush[2], Asaf Nissenbaum[2], Raz Gvishi[2], Galit Bar[2], Igor Lubomirsky[1], David Ehre[1*]

[1]Department of Molecular Chemistry and Materials Science, Weizmann Institute of Science, Rehovot, 761001, Israel

[2]Soreq NRC, Yavne 81800, Israel

[‡]These authors contributed equally: Tali Pechersky-Savich, Guy Lazovski

*Authors to whom correspondence should be addressed: david.ehre@weizmann.ac.il, guyla@soreq.gov.il

Section S1. 3D printed fixture for impedance measurements

The measurement fixture has been described in detail in Ref.[1]. The fixture was printed using Filamentech™ ABS on a Flashforge™ Adventurer 3 (wall thickness 0.8mm, fill density 15%, pattern-hexagons) 3D printer. A disc-shaped aluminum electrode, diameter 25.4 mm, was attached with cyanoacrylate adhesive to a vertical rod, which can be moved to adjust to the sample thickness. A second Al disc electrode was glued to the bottom stage. The rod, through which the longest wire runs, was coated with silver paint and grounded to a sub-miniature version A (SMA) connector with a copper wire, effectively enclosing the silver-coated wire in a Faraday cage. The connecting wires for the electrodes were glued with silver epoxy to the aluminum surface and soldered at the opposite end with Sn-Ag-Cu (SAC) alloy to the SMA connectors (**Figure S1**).

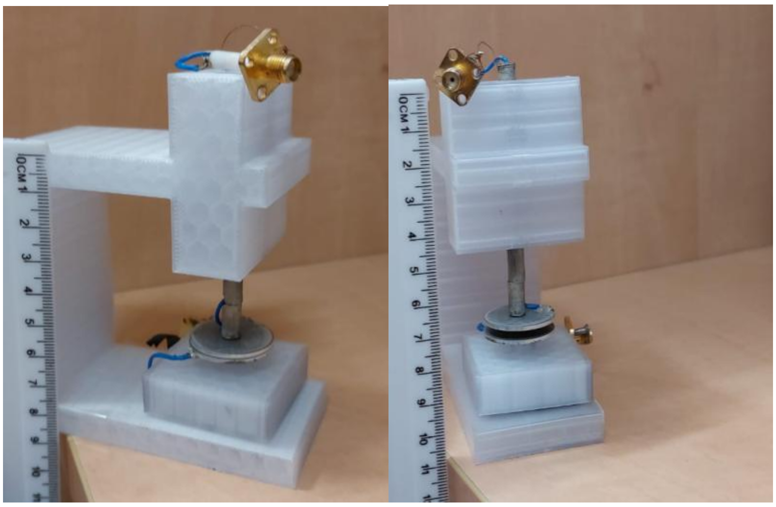

Figure S1. Two views of the 3D printed ABS measurement fixture. Disc-shaped Al electrodes, diameter 25.4 mm, are connected to sub-miniature version A (SMA) BNC connectors using Sn-Ag-Cu (SAC) solder alloy. The ABS rod is coated with silver paint and can be moved vertically to adjust the electrode spacing to the sample thickness.[1]

Section S2. Protocol for preparing TMOS-based aerogels using hot supercritical drying in pure acetonitrile

Table S1. Gram weight of components and time required for preparation of TMOS-based aerogel discs prior to hot super critical drying.

Measured mass density (mg/cm³)	Solution A (g.)		Mixing time (min)	Solution B (g.)			Mixing time (min)	Mixing of incorporated solution (min)	Gelation time (min)
	Ethanol	TMOS		H_2O	NH_4OH solution	Ethanol			
10	3.65	0.32	5	0.32	0.6	3.65	6	1	40
45	3.27	0.93	5	0.84	0.442	3.27	6	1	30
60	2.93	1.11	5	1.33	0.145	2.93	6	1	42
70	2.87	1.3	5	1.56	0.128	2.87	6	1	31
80	2.64	1.4	5	1.67	0.11	2.64	6	1	17
100	2.71	1.88	5	1.71	0.087	2.71	6	1	21
120	2.60	2.28	5	1.64	0.067	2.60	6	1	24
140	2.48	2.66	5	1.57	0.04	2.48	6	1	25
150	2.64	3.4	5	1.67	0.03	2.64	6	1	42
170	2.17	3.39	5	1.70	0.019	2.17	6	1	44
190	1.92	3.89	5	1.81	0.017	1.92	6	1	48
200	1.74	4.23	5	1.98	0.015	1.74	5	1.5	42

Table S2. Molar ratios of components input for synthesis of TMOS aerogel discs characterized by theoretical density, volumetric shrinkage and measured density.

Measured density (mg/cm^3) / Precursor	10	45	60	70	80	100	120	140	150	170	190	200
TMOS	1	1	1	1	1	1	1	1	1	1	1	1
Ethanol (Sol. A)	37.769	11.633	8.725	7.294	6.232	4.748	3.772	3.081	2.566	2.111	1.636	1.356
Ethanol (Sol. B)	37.769	11.633	8.725	7.294	6.232	4.748	3.772	3.081	2.566	2.111	1.636	1.356
H_2O	22.967	11.310	11.144	10.850	10.632	8.036	6.328	5.119	4.219	4.284	3.977	3.983
NH_4OH	0.712	0.180	0.049	0.037	0.027	0.017	0.011	0.007	0.003	0.002	0.002	0.001
Gel. Time (min)	40	30	30	31	17	20.5	24	24.5	42	44	48	42
Shrinkage‡	-48	2	10	15	18	15	14	13	12	14	10	9

[1] Shrinkage is the measured % change in volume of the aerogels following hot super critical drying (SCD) in pure acetonitrile.

Table S3. Dimensions of aerogel discs following hot super critical drying.

Sample	Thickness (mm)	Density (mg/cm^3)	Diameter (mm)
AGP730	2.83	79.88	28.29
AGP731	2.91	80.26	28.50
AGP732	2.84	215.00	28.13
AGP729	2.85	64.92	28.43
AGP736	2.91	146.37	29.05
AGP818	2.50	21.45	29.92
AGP820	2.52	19.76	29.53

Table S4. Dimensions of aerogel discs, following hot super critical drying, used for UV-VIS-IR spectroscopy.

Sample name	Mass density (mg/cm^3)	Thickness (mm)
AGP265	190	4.83
AGP234	150	4.87
AGP229	120	4.85
AGP256	80	4.63
AGP216	45	4.92
AGP239	10	5.71

Section S3. SEM imaging of TMOS-based aerogels

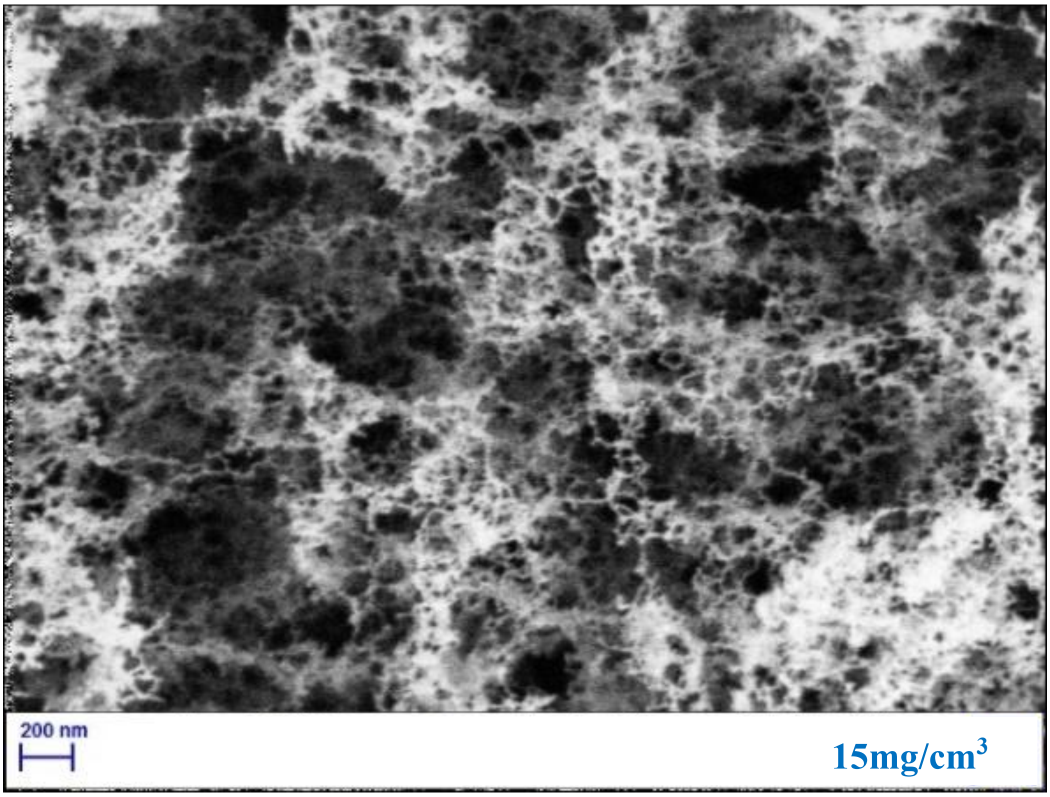

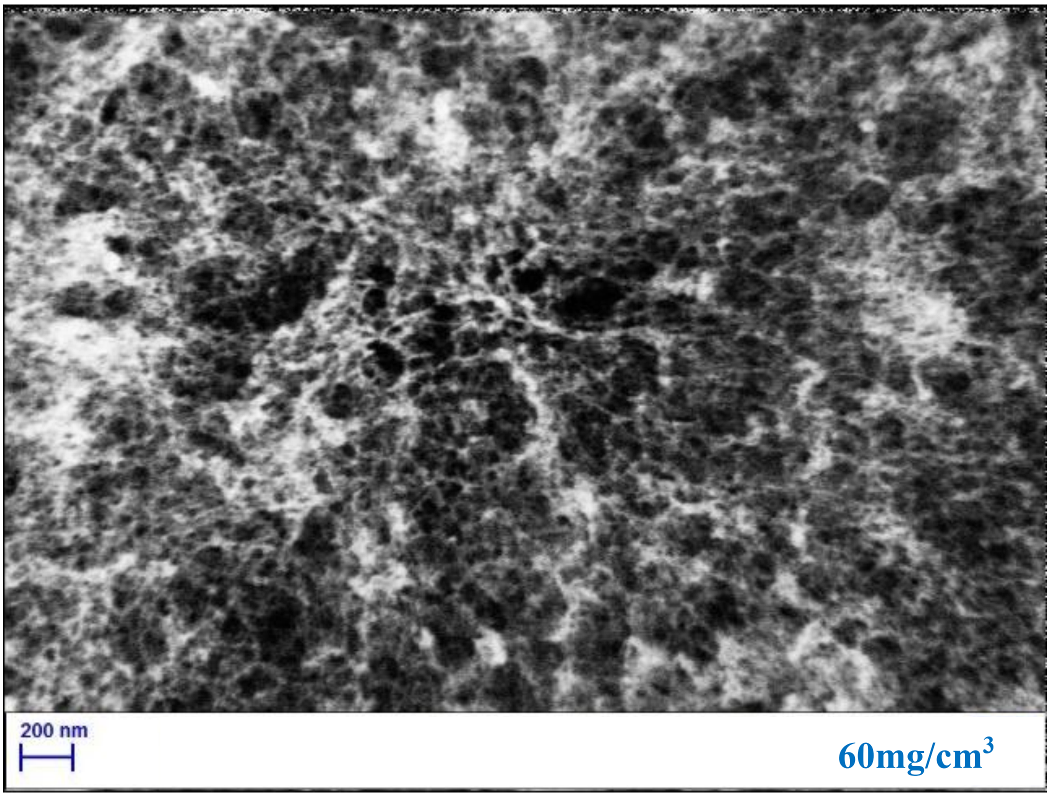

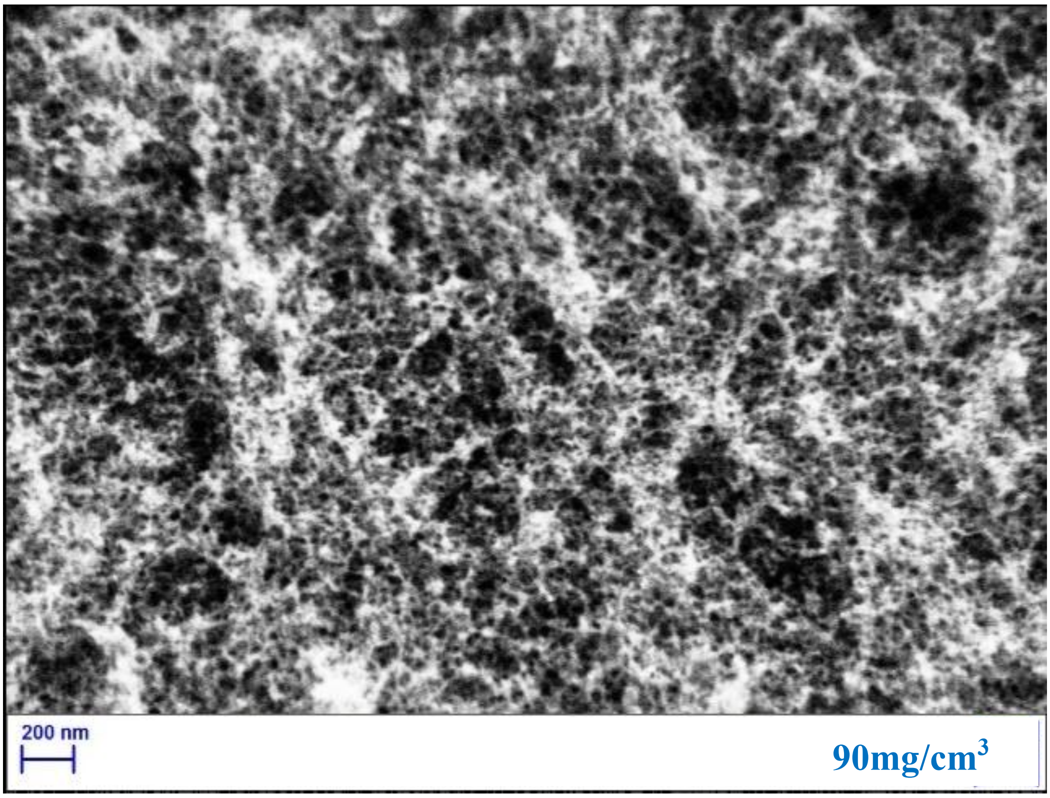
200 nm
90mg/cm3

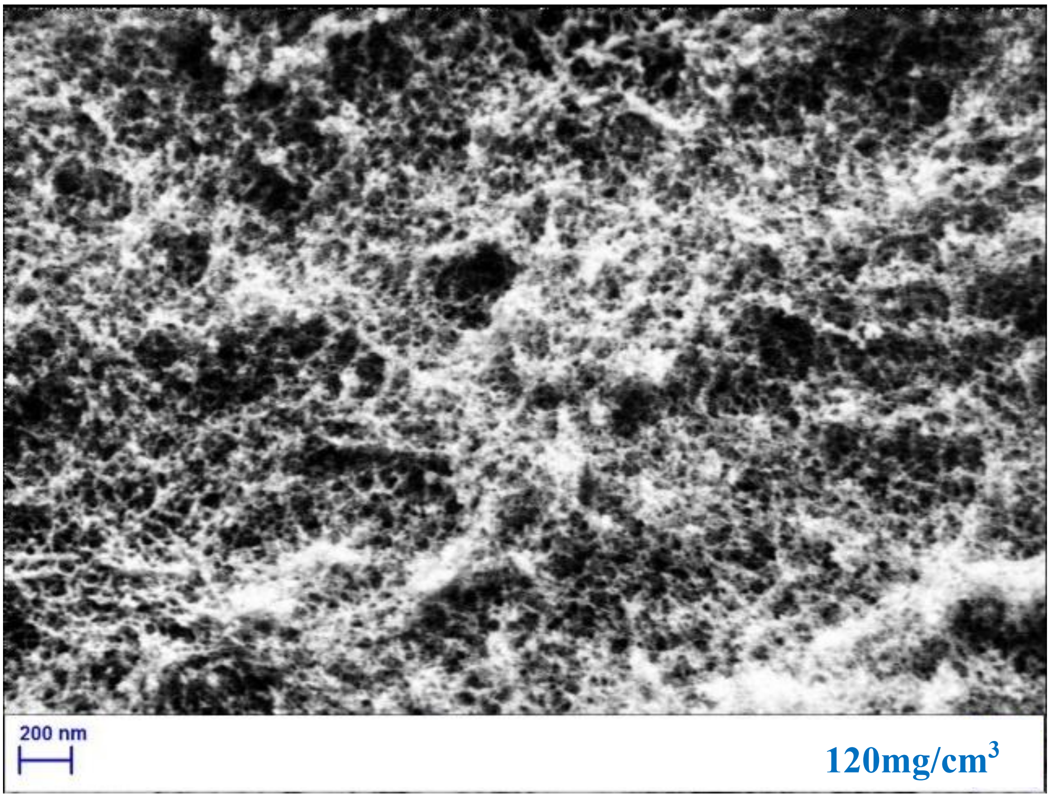
200 nm
120mg/cm3

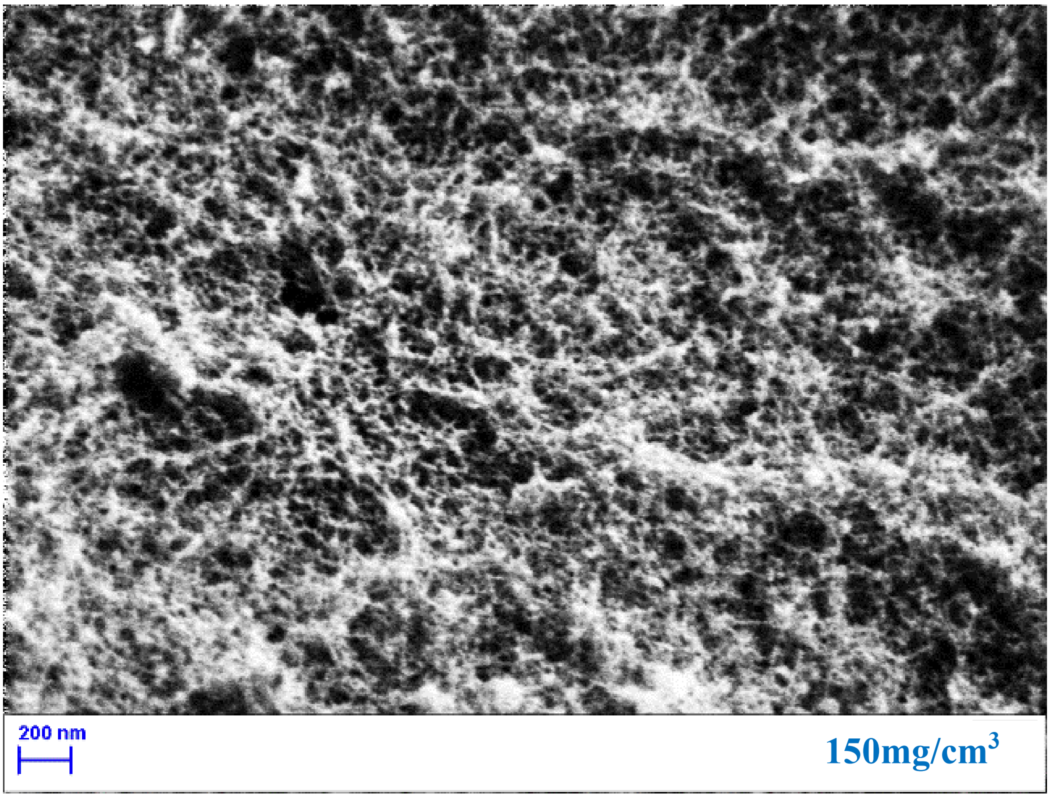

Figure S2. SEM images of TMOS-based aerogels, synthesized as described in Materials and Methods section of the main text, with mass density ranging from 15 to 150 mg/cm^3. Scale bar indicates 200 nm.

Section S4. Impedance measurements of TMOS-based aerogels in the VLF frequency range

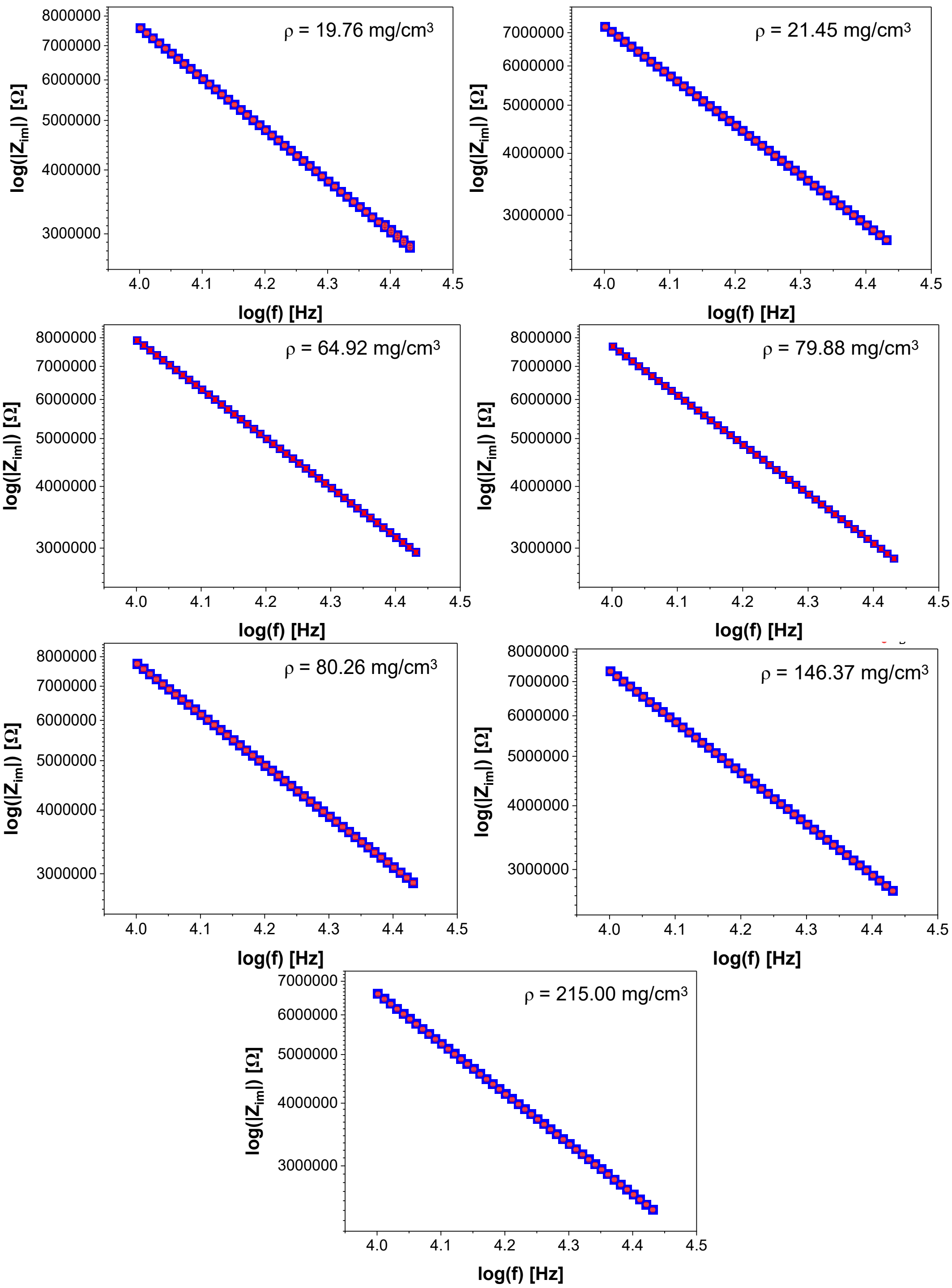

Figure S3. Bode plots of Z_{im} (complex impedance data measured in duplicate) for TMOS-based aerogels in dry oxygen, mass density 19.76 mg/cm^3 to 215 mg/cm^3. Extrapolating the linear fit to the y-axis provides the capacitance of the aerogel sample (Eq. 7 in the main text), uncorrected for the fringing field (Eq. 8 in the main text).

Table S5. Electrostatic charging of aerogel disc samples.

Mass Density [mg/cm^3]	Sample charge before insertion into the O_2 filled zip-lock bag	Sample charge following 15min in the O_2 filled zip-lock bag	Sample charge following impedance measurements in O_2 in the zip-lock bag
34.36	-0.21V	+2.2V	+1.0V
41.1	-1.1V	+3.6	+1.5

References

1. Pechersky Savich, T.; Varenik, M.; Wachtel, E.; Lazovski, G.; Lubomirsky, I.; Ehre, D., 3D-printed fixture for capacitance measurements of very low permittivity dielectrics. *Review of Scientific Instruments* **2025,** *96* (3).
